# How do researchers conceptualize and plan for the sustainability of their NIH R01 implementation projects?

**DOI:** 10.1186/s13012-019-0895-1

**Published:** 2019-05-09

**Authors:** Alekhya Mascarenhas Johnson, Julia E. Moore, David A. Chambers, Jennifer Rup, Camellia Dinyarian, Sharon E. Straus

**Affiliations:** 1 Li Ka Shing Knowledge Institute, St. Michael’s Hospital, Toronto, ON Canada; 2 Division of Cancer Control and Population Sciences, National Cancer Institute, National Institutes of Health, Bethesda, MD USA; 3 University of Toronto, Toronto, ON Canada

**Keywords:** Sustainability, Implementation, Frameworks

## Abstract

**Background:**

Inadequate sustainability of implementation of evidence-based interventions has led to calls for research on how sustainability can be optimized. To advance our understanding of intervention sustainability, we explored how implementation researchers conceptualized and planned for the sustainability of their implemented interventions with studies funded by the United States (US) National Institutes of Health (NIH).

**Methods:**

We used sequential, mixed methods to explore how researchers conceptualized and planned for the sustainability of the health interventions using (1) a document review of all active and completed US NIH R01 Grants and Equivalents reviewed within the Dissemination and Implementation Research in Health (DIRH) Study Section between 2004 and 2016 and (2) a qualitative content analysis of semi-structured interviews with NIH R01 DIRH grant recipients.

**Results:**

We found 277 R01 profiles within the DIRH study section listed on the US NIH RePORTER website including 84 that were eligible for screening. Of the 84 unique projects, 76 (90.5%) had primary implementation outcomes. Of the 76 implementation project profiles, 51 (67.1%) made references to sustainability and none referred to sustainability planning. In both profiles and interviews, researchers conceptualized sustainability primarily as the continued delivery of interventions, programs, or implementation strategies. Few researchers referenced frameworks with sustainability constructs and offered limited information on how they operationalized frameworks. Researchers described broad categories of approaches and strategies to promote sustainability and key factors that may influence researchers to plan for sustainability, such as personal beliefs, self-efficacy, perception of their role, and the challenges of the grant funding system.

**Conclusions:**

We explored how US NIH R01 DIRH grant recipients conceptualized and planned for the sustainability of their interventions. Our results identified the need to test, consolidate, and provide guidance on how to operationalize sustainability frameworks, and to develop strategies on how funders and researchers can advance sustainability research.

**Electronic supplementary material:**

The online version of this article (10.1186/s13012-019-0895-1) contains supplementary material, which is available to authorized users.

Contributions to the literature

- Research gaps exist in how to optimize sustainability of implementation of evidence-based interventions.
- We found that funded implementation researchers vary in their definitions of sustainability and their use of sustainability frameworks.
- We identified strategies for funders to consider to advance the sustainability research agenda in implementation science.

## Background

Despite research advances to narrow the research to practice gaps, the sustainability of evidence-based health interventions in clinical and community settings remains a significant challenge [1, 2]. Knowledge syntheses show that sustainability of evidence-based interventions is often ignored and this gap has been identified as one of the most critical gaps in implementation science [2–7]. Little is known about how to sustain effective interventions, and the failure to sustain has implications for health system costs, patient outcomes, and support for future implementation work [8].

There have been calls for research on how sustainability can be defined, planned for, and considered in implementation research [2, 9]. In particular, it is unclear whether implementation researchers consider sustainability when planning or executing their projects. As such, we conducted a sequential, mixed methods study to explore how implementation researchers funded by the United States (US) National Institutes of Health (NIH) conceptualized and planned for intervention sustainability as this has not been reported previously. We explored how do the US NIH-funded implementation researchers (1) define sustainability, (2) use sustainability frameworks in their implementation projects, and (3) plan for sustainability of interventions and (4) what influences these implementation researchers to plan for sustainability? We perceived these successfully funded implementation researchers to be experts in this field and would provide insights into the state of the science.

## Methods

### Phase 1: Document review

#### Data source

The NIH is one of the world’s largest public funders of health research, funding research within the US and globally [12, 13]. It funds studies focused on the implementation of evidence-based interventions, which are primarily reviewed through the Dissemination and Implementation Research in Health (DIRH) Study Section [13]. We conducted a document review of all active and completed US NIH R01 Grants and Equivalents (the mechanism used to fund implementation research) reviewed within the DIRH Study Section using the NIH Research Portfolio Online Reporting Tools, Expenditures and Results (RePORTER) Database. The NIH RePORTER Database is an online repository of NIH-funded research project profiles [14]. We then completed a PubMed search for publications on these studies and abstracted data on whether sustainability was planned for or measured.

**Fig. 1:**
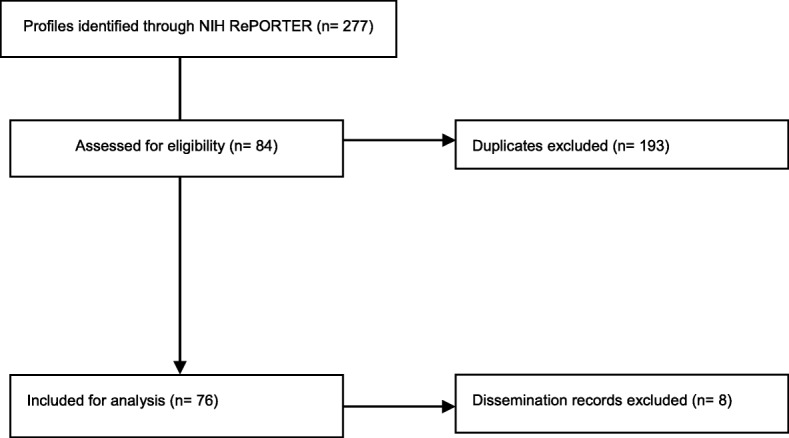
Flow diagram of identification, screening, and inclusion of project profiles for analysis. Between September 19 and October 05, 2016, we exported data from all R01 Grants and Equivalent project profiles listed in the NIH RePORTER Database reviewed by the DIRH study section. We removed duplicate project profiles with the same grant identification number. Two analysts independently screened project profiles and excluded profiles that did not have primary outcomes related to implementation. We defined implementation as the use of strategies to adopt and integrate evidence into practice within specific settings [15]. We only included projects with an implementation focus for further data abstraction and excluded those that had primary outcomes focused on dissemination. We defined dissemination as the targeted distribution of evidence to a specific public health or clinical practice audience (e.g., the comparative the effectiveness of two modes of delivering information to target audiences) [1]. Of the 277 active or completed R01 projects reviewed by the DIRH study section listed on the NIH RePORTER website, 84 were eligible for screening. These projects had start dates ranging from September 1, 2004, to August 25, 2016. Of the 84 projects, 76 (90.5%) had primary implementation outcomes and 8 (9.5%) had only dissemination outcomes. Analysts abstracted descriptive data from the 76 included project profiles, and inter-rater reliability was 82%. We then completed PubMed searches using the investigators’ names to identify articles related to the funded project. We identified 47 relevant articles and abstracted data from these on whether sustainability was addressed, planned for, and/or measured. Two people abstracted data independently, and kappa was calculated [15, 16]

Details on data collection and analysis [15, 16] can be found in Fig. 1.

### Phase 2: Qualitative content analysis

We conducted semi-structured interviews (Additional file 1: Appendix A) with principal investigators (PIs) identified in phase 1 to explore their sustainability definition, use of frameworks, strategies to plan for sustainability, and what influenced their decision to plan for sustainability. We sent email invitations to all phase 1 PIs with reminders every 2 weeks. Further details on recruitment and analysis [17–22] can be found in Additional file 2: Appendix B. Response rate to email invitations was 14.5% (*n* = 11).

We did a directed content analysis [19] of the interview transcripts. Analysts conducted two rounds of double coding on 40% of transcripts (*n* = 4) until kappa coefficients exceeded 0.60 [22]. Analysts exported data under the “definition” code and mapped it to the five concepts included in a published sustainability definition [10]. The definition was used to clarify what constructs existed in PIs’ conceptualizations of sustainability.

### Integration of phase 1 and phase 2 results

Qualitative and quantitative data from each phase were integrated narratively, and the results are presented using a contiguous approach [23].

## Results

### Phase 1: Document review

See Fig. 1 for results of identification, screening, and abstraction.

#### Description of funded implementation projects

The NIH funded 277 projects reviewed by the DIRH study section between 2004 and 2016, and 76 met the inclusion criteria for our study (i.e., they were implementation projects). The projects received a mean of $1,896,260 USD (*SD* = $1,034,716; range $328,791 to $5767242) in funding. The average length of projects was 4.8 years (*SD* = 1.4; range 1.3 to 12.6 years). The majority of projects focused on implementation in either clinical (*n* = 24, 31.6%) or community settings (*n* = 24, 31.6%) (Table 1) and included analysis of clinical or health-related outcomes (*n* = 49, 64.5%). A total of 47 published manuscripts describing 44 unique projects were reviewed for supplementary information; 38 stated plans to consider sustainability, and 32 proposed to measure it.

**Table 1:** Setting of implementation projects

Implementation	Implementation projects	
	(*n*)	(%)
Chronic care hospital	3	3.9
Clinic	24	31.6
Community	24	31.6
Government	2	2.6
Home	2	2.6
Acute care hospital	12	15.8
Workplace	1	1.3
School	1	1.3
Online social network	1	1.3
Not reported	6	7.9
Total	76	100

#### Conceptualization of sustainability in implementation project profiles

Of the 76 implementation projects, 51 (67.1%) included sustainability synonyms or variants in their NIH RePORTER profiles. The words sustainability (*n* = 22, 22.9%), sustained (*n* = 9, 9%), and maintenance (*n* = 14, 14.5%) were the most commonly used terms (Additional file 3: Appendix C). References to sustainability (Table 2) included descriptions of their intent to evaluate the impact of a strategy on the sustainability of evidence-based programs (EBPs) or practices (*n* = 11, 18.3%); measure the sustainability of EBPs, strategies, practices, or health outcomes (*n* = 10, 16.6%); examine factors associated with sustainability of interventions during or after implementation (*n* = 13, 21.6%); estimate intervention sustainability costs and benefits (*n* = 1, 1.7%); and determine elements of EBPs to enhance sustainability (*n* = 1, 1.7%). Only three NIH RePORTER profiles (3.9%) described the intent to study sustainability exclusively. A few profiles referred specifically to the sustainability of their research study by describing the potential large-scale impact or public health relevance of their work (*n* = 7, 11.7%) or describing their intent to disseminate results to promote sustainability (*n* = 3, 5.0%). Profiles referred to implementation frameworks with sustainability constructs; however, none mentioned sustainability frameworks. The Reach, Effectiveness, Adoption, Implementation, Maintenance (RE-AIM) framework (*n* = 11, 14.5%) was the most frequently referenced framework. One profile (1.3%) referenced the Exploration, Preparation, Implementation, Sustainment (EPIS) framework, and one (1.3%) referred to the Replicating Effective Programs (REP) framework.

**Table 2:** Frequency of reasons for sustainability references across R01 DIRH implementation project profiles

Reason for referring to sustainability	Frequency (*n*)	Percentage (%)
Intent to examine factors associated with sustainability during or after implementation	13	21.6
Intent to evaluate the impact of a strategy on sustainability of programs or practices	11	18.3
Intent to measure sustainability (e.g., sustained delivery of programs or implementation strategies, sustained health outcomes, or sustained practices)	10	16.6
Public health relevance statement or hypothesis of how work will impact sustainability of EBPs	7	11.6
Describing need for better implementation supports/strategy development	5	8.3
Reference to evidence that intervention has produced sustained health or behavioral outcomes	4	6.6
Intent to disseminate of R01 results to promote sustainability of EBPs in practice	3	5.0
Intent to evaluate comparative sustainability of each strategy	2	3.3
Reference to stakeholder engagement and impact on sustainability	2	3.3
Intent to sustain implementation efforts with capacity building and leadership development	1	1.6
Intent to estimate sustainability costs and benefits for an intervention	1	1.6
Intent to determine elements of programs that will enhance sustainability	1	1.6
Total	60	100

### Phase 2: Qualitative content analysis

#### Interviewee demographics

Interviewee demographics are outlined in Table 3. Two (18.1%) of the 11 interviewees included references to sustainability in their NIH RePORTER profiles.

**Table 3:** Characteristics of interviewees

Interviewee ID	Unit of analysis	Researcher setting
62	Organization	Academic research institute
22	Individual	Academic research institute
11	Organization	Academic research institute
10	Organization	Academic research institute
12	Organization	Non-profit research institute
24	Individual	Academic research institute
026	Organization	Academic research institute
044	Community	Academic research institute
050	Community	Academic research institute
09	Organization	Academic research institute
75	System	Non-profit research institute

#### How do R01 implementation researchers define sustainability?

Interviewees provided very different sustainability definitions (Table 4), although nearly all defined sustainability as the continued delivery of evidence-based intervention implementation strategies within an organization or system (Table 5). Interviewees emphasized that the sustained intervention, program, or policy must have proven effectiveness in its original implementation setting. They said the reach of the implementation strategy must remain consistent over time, especially after researchers exit the project.

**Table 4:** Interviewee definitions of sustainability mapped to a comprehensive definition of sustainability [10]

Interviewee ID	After a defined period of time	A program, clinical intervention, and/or implementation strategies continue to be delivered	Individual behavior change (i.e., clinician, patient) is maintained	The program and individual behavior change may evolve or adapt	Continuing to produce benefits for individuals/systems
62	X	X			
22		X	X	X	X
11		X			
10		X			
12	X	X		X	
24	X				X
026		X			
044	X	X			
050		X			
09	X	X			
75	X	X		X	X

**Table 5:** Participant comments on sustainability definitions

“ … continued and ongoing use of implementation strategies that work to improve adoption of guidelines”—22	
“ … ability of the organization in which the evidence-based program that has been implemented to maintain its offering at a high or desired level of fidelity”—10	
“Having the intervention be able to sort of stand-alone if you will, either online with some sort of a company or some sort of a system that would allow it to remain online or publication of sort of the manual”—11	

One interviewee defined sustainability as individuals’ maintenance of practices. Three interviewees specified that sustainability necessitates adaptations to intervention implementation strategies to changes in political climate, funding, legislation, health and social service system, organizational or community needs, and staffing. Three interviewees described maintaining improved intervention outcomes as a key sustainability definition construct. In addition to the five constructs from the definition, one interviewee included the development of organizational infrastructure to deliver the implementation strategy as a sustainability definition construct. Another interviewee mentioned that sustainability includes the intervention becoming a product that can be scaled up or survive as a business (Table 5).

#### How do R01 implementation researchers use sustainability frameworks?

Participants mentioned using the following frameworks:

- Consolidated Framework for Implementation Research (CFIR) (*n* = 3)
- Scheirer and Dearing framework (*n* = 2)
- Framework for dissemination by Peter Mendel and colleagues (*n* = 1)
- EPIS framework (*n* = 2)
- Dynamic Sustainability Framework (DSF) (*n* = 2)
- RE-AIM (*n* = 2)
- Plan, Do, Study, Act (PDSA) (*n* = 1)
- REP Framework (*n* = 1)

Interviewees found it challenging to describe how to operationalize the frameworks to conceptualize sustainability in implementation research; however, they outlined three broad approaches. One approach was to develop a stakeholder interview guide using sustainability constructs from a framework. These interviews then provided information on how the sustainability constructs were present in a given setting; these data were then used to inform sustainability-related decisions at project onset. A second approach was to use the framework to brainstorm what challenges might hinder sustainability, and then use conjoint analysis to determine what challenges were most important or feasible to address. The top-ranked challenges were then mapped to implementation strategies; the strategies may not have been specific to sustainability. In both of these approaches, stakeholders and implementation teams were consulted. The third approach used frameworks to develop surveys or interviews for sustainability evaluations. Research teams reviewed the constructs in each framework and then selected, by consensus, what constructs would be measured in their project.

#### How do R01 implementation researchers plan for sustainability?

Few participants said they had a sustainability plan. Of those who had plans, two main approaches emerged: the “weaning off” approach and the “strategic funding approach.” These approaches targeted the sustainability of evidence-based interventions within host settings. Both approaches began at implementation onset.

In the “weaning off” approach, research personnel built capacity among front-line implementers to assume the role of implementation leaders after the research personnel left the setting. As implementation progressed, front-line implementers were encouraged to step into leadership using a graduated approach. Research personnel remained available for coaching and eventually exited from the setting.

In the “strategic funding” approach, implementation researchers used research funds only for research activities, such as PI time and participant recruitment. Prior to implementation, researchers secured buy-in from host organizations to fund implementation. Front-line implementers assumed the implementation activities.

All interviewees described some general strategies that they used to plan for sustainability (Table 6). The majority of these strategies were targeted towards the sustainability of evidence-based interventions within organizations, communities, or systems and not individuals.

**Table 6:** General sustainability planning strategies

Category	Description
System-level stakeholder buy-in	Advocate for the development of policies that encourage the use of the intervention/program/practice both before and during implementation. Network with professional organizations that could either promote the intervention/program/practice or incorporate the intervention/program/practice as part of their larger professional curriculum. Organize ongoing stakeholder consultations to monitor changes in the context that could require adaptations or to advocate for more funding
Organizational/community-level stakeholder buy-in	Encourage leaders to buy-in and talk about the intervention/program/practice regularly in staff meetings. Consult with individuals implementing the intervention to find out if the proposed intervention/program/practice will be sustainable after research funding is removed (e.g., site visits, formative evaluations) Co-develop implementation strategies with stakeholders
Organizational incentives	Build in program indicators into performance reviews, organization leaders also build other incentives for employees to use the intervention/program/practice and they document their progress
Staff-turn over packages	Generate new staff orientation and training packets so that new hires learn about the intervention/program/practice as soon as they are on boarded
Capacity building at all levels (organization, community, system)	Link implementation teams to a resource package or other organizations that provide ongoing training. Provide implementers with a free social media tool/learning collaborative where they can have fast access to resources and connect with other implementers
Organization-level continuous quality improvement	Host organization staff that are trained to use PDSA cycles to monitor the fit of the intervention/program/practice, anticipate challenges, and adapt where needed over time
Intervention monetization	Publish a training handbook that can be purchased at a popular book store. Sell online resources to prospective implementers (e.g., one-time fee for unique log-in)
Guidance from intervention developers	Provide guidance on what the core and what the kind of adaptable periphery of the intervention/program/practice, so that when changes need to be made implementers have a sense of what key elements need to be sustained
Programmatic approach to research	Ensure each implementation study has elements of sustainability (e.g., one arm gets early sustainability planning) that can be followed up on in subsequent studies and the funding for the intervention/program/practice continues

#### What influences R01 implementation researchers’ decision to plan for sustainability?

Interviewees described various factors that may influence researchers’ decisions to plan for sustainability both at the individual level and in the surrounding environment.

##### Individual level—researcher beliefs about sustainability, self-efficacy, and perception of their role

Interviewees who self-identified as having a special interest in sustainability research said that their motivation came from observing the harm that research personnel can do if they leave a setting without making any sustainability plans. Some interviewees said that they were hesitant to plan for sustainability because they had insufficient expertise or knowledge on it. Others suggested that many implementation researchers do not consider sustainability planning as their role.

sustainability is something that we researchers rarely think much about, I mean we write grants, we say at the end, and at the end of this study we will publish in leading journals. The assumption there is that somebody will do something then. And it may be a fallacious assumption, it frequently is —050

##### Environmental context—intervention characteristics and grant funding system

Some interviewees said that their capacity for sustainability planning depended on whether they could design a “step down” approach or a “leaner phase” in the final study year so that the program or intervention is left in the hands of the host organization. Interviewees said that the grant funding system has a significant influence on their decision and ability to plan for sustainability. They found it challenging to measure sustainability within the time frame of an R01 grant and suggested ideas for funding agencies to encourage sustainability planning (Table 7).

**Table 7:** Participant suggestions for implementation research funders

*Include the requirement to plan for sustainability in program announcements*: This was described as similar to the announcements from the Centre of Disease Control and Prevention. Current NIH announcements include language about studying sustainability but not about planning.	
*Request for proposals (RFP) could include sustainability requirements*: Applicants should be required to list sustainability outcome measures (e.g., sustainability 6 months post implementation).	
*Offer a supplement OR non-competitive renewal specifically for sustainability*: This could include parameters such as the requirement to use funds only for evaluation or testing of lean, internally resourced strategies for sustainability.	
*Deliver funding in phases so that there is funding specifically allocated for sustainability efforts*: This ensures that there is money for sustainability and full funding is not received until it is clear that the program has the potential capacity to be sustained.	
*Include the requirement to engage stakeholders and demonstrate the need for this program/intervention in the relevant setting*: Applicants must describe to what extent the work is a priority for the context and to what extent the relevant stakeholders have been involved in the planning and evaluation process.	

## Discussion

Over the past 12 years, the US NIH invested almost $145 million USD in implementation projects reviewed within the DIRH study section. Of these projects, 51 (67.1%) referenced sustainability in their RePORTER profiles. Both researcher interviews and RePORTER profiles revealed that implementation researchers conceptualized sustainability primarily as the continued delivery of implementation strategies. This result highlights the lack of clarity around sustainability definitions as researchers and implementers must consider if they are sustaining the evidence-based intervention, the implementation strategy, the behavior change, and/or the outcomes of these behavior changes. Adaptation was not substantively described in the interviews. Interviewees described broad categories of approaches for sustainability. They described key factors that may influence researchers to create a plan such as personal beliefs, self-efficacy, perception of their role, and the challenges of the US grant funding system.

The use of frameworks, theories, and models is a key challenge within implementation science, [24–27] particularly the lack of the use of sustainability frameworks [2]. A recent review revealed identified 62 sustainability models, checklists, tools, processes, strategies, conceptualizations, and frameworks [11]. This complexity may explain why few researchers in our study referred to sustainability frameworks. Rather than developing new sustainability frameworks, there is a need to generate knowledge on how to apply their constructs to advance sustainability research and to make it easy for researchers to select and apply them.

Our study results raise an interesting question about who is responsible for sustainability planning. Sustainability is a dynamic process that involves complex interactions between interventions, practice settings, and a broader ecological system [28]; therefore, sustainability planning should involve a dynamic, multifaceted approach with the involvement of all those who have a stake in sustainability such as funders, researchers, practitioners, and program beneficiaries.

The grant funding systems in many countries do not contain many incentives to study sustainability, and the time frame of grants poses a disincentive for those who want to measure sustainability. Our findings identified some strategies that funders can consider to enhance sustainability, such as including a requirement to plan for sustainability within program announcements and offering non-competitive renewals for sustainability. We also found areas where researchers can strengthen their implementation and sustainability research grants. Researchers who describe their intentions to report and measure multiple constructs of sustainability and those who test sustainability planning strategies may appear creative, which may optimize grant success. Researchers may also benefit from fostering partnerships with implementation practitioners to understand the long-term impact of their work and the value of sustainability planning and to exchange knowledge on what influences sustainability. These findings on the US NIH R01 grant scheme can be useful for funders and researchers worldwide who are involved in similar operating grant schemes. Some examples of schemes that fund implementation work outside the US are the project grants from the Canadian Institutes for Health Research [29] and the Invention for Innovation grants from the National Institute for Health Research [30].

### Limitations

First, our study only examined the information available on the US NIH RePORTER profiles and published protocols. This information does not provide complete descriptions of DIRH-funded implementation projects, nor does it include implementation studies that were reviewed elsewhere at the NIH. It is possible that information on sustainability may be documented elsewhere such as in final study publications. For this reason, we decided to conduct interviews with funded researchers. Second, our study focused exclusively on implementation researchers funded by the US NIH. There may be additional insights that can be gleaned from doing a similar study of implementation work funded by other agencies in different countries; however, the NIH is one of the world’s largest funders of health evidence implementation research. Third, due to our small sample size and poor interview response rate, the interview findings may not be generalizable to all implementation researchers. Interviewee demographics reflected that they were from different institutions; however, it is possible that key insights are missing and that there was a bias in those participating in interviews, especially since most interviewees had mentioned sustainability in their project profiles. Qualitative research studies often include this limitation. The goal of qualitative research is to provide rich description of a phenomenon, not to generate perceptions that are representative of the general population from which the study sample was drawn [19]. Fourth, there may be different arguments on whether it is always appropriate for NIH-funded implementation researchers to consider sustainability. Some sustainability experts perceive sustainability as a process that should be considered at the onset of all implementation work, and others perceive sustainability as an outcome that should be considered once the intervention has been evaluated [9]. There may be implementation projects with findings that should not be sustained. There is also a tension between sustainability and adaptation. Our study does not expound on this debate. Despite these limitations, we believe that this study adds new information to the sustainability discourse. This is the first study that we are aware of that examined researcher perspectives on sustainability conceptualization and planning.

## Conclusions

Implementation researchers varied in their conceptualization and planning for sustainability within their funded implementation projects. Our results identified the need to provide guidance on how to identify, select, and use sustainability frameworks and to develop strategies on how funders and researchers can advance sustainability research.

## Additional files

Additional file 1: Appendix A: appendix representing the questions used for the semi-structured interview guide. (DOCX 15 kb)

Additional file 2: Appendix B: appendix representing further details on the data collection and analysis for the document review and the qualitative interviews. (DOCX 18 kb)

Additional file 3: Appendix C: appendix representing the frequency of sustainability synonyms used across R01 DIRH implementation project profiles. (DOCX 14 kb)

## Abbreviations

CFIR: Consolidated Framework for Implementation Research
DIRH: Dissemination and Implementation Research in Health
DSF: Dynamic Sustainability Framework
EBPs: Evidence-based programs
EPIS: Exploration, Preparation, Implementation, Sustainment
NIH: National Institutes of Health
PDSA: Plan, Do, Study, Act
PI: Principal investigator
RE-AIM: Reach, Effectiveness, Adoption, Implementation, Maintenance
REP: Replicating Effective Programs
RePORTER: Research Portfolio Online Reporting Tools, Expenditures and Results
RFP: Request for proposals

## References

[1] Glasgow R, Chambers D (2012). Developing robust, sustainable, implementation systems using rigorous, rapid and relevant science. Clin Transl Sci.

[2] Proctor E, Luke D, Calhoun A, McMillen C, Brownson R, McCrary S, Padek M. Sustainability of evidence-based healthcare: research agenda, methodological advances, and infrastructure support. Implement Sci. 2015;10:1–13. 10.1186/s13012-015-0274-5 PMC4494699 26062907

[3] Wiltsey Stirman S, Kimberly J, Cook N, Calloway A, Castro F, Charns M. The sustainability of new programs and innovations: a review of the empirical literature and recommendations for future research. Implement Sci. 2012;7:1–19. 10.1186/1748-5908-7-17 PMC3317864 22417162

[4] Ament S, de Groot J, Maessen J, Dirksen C, van der Weijden T, Kleijnen J (2015). Sustainability of professionals’ adherence to clinical practice guidelines in medical care: a systematic review. BMJ Open.

[5] Iwelunmor J, Blackstone S, Veira D, Nwaozuru U, Airhihenbuwa C, Munodawafa D, Kalipeni E, Jutal A, Shelley D, Ogedegbe G. Toward the sustainability of health interventions implemented in sub-Saharan Africa: a systematic review and conceptual framework. Implement Sci. 2016;11:1–27. 10.1186/s13012-016-0392-8 PMC4804528 27005280

[6] Scheirer M (2005). Is sustainability possible? A review and commentary on empirical studies of program sustainability. Am J Eval.

[7] Gould D, Moralejo D, Drey N, Chudleigh J, Taljaard M. Interventions to improve hand hygiene compliance in patient care. Cochrane Database Syst Rev. 2017;9:1–109. 10.1002/14651858.CD005186.pub4 PMC6483670 28862335

[8] Rankin N, Butow P, Thein T, Robinson T, Shaw J, Price M, Clover K, Shaw T, Grimison P. Everybody wants it done but nobody wants to do it: an exploration of the barrier and enablers of critical components towards creating a clinical pathway for anxiety and depression in cancer. BMC Health Serv Res. 2015;15:1–8. 10.1186/s12913-015-0691-9 PMC4307637 25608947

[9] Shelton Rachel C., Cooper Brittany Rhoades, Stirman Shannon Wiltsey (2018). The Sustainability of Evidence-Based Interventions and Practices in Public Health and Health Care. Annual Review of Public Health.

[10] Moore J, Mascarenhas A, Bain J, Straus S. Developing a comprehensive definition of sustainability. Implement Sci. 2017;12:1–8. 10.1186/s13012-017-0637-1 PMC5581411 28865479

[11] Lennox L, Maher L, Reed J. Navigating the sustainability landscape: a systematic review of sustainability approaches in healthcare. Implement Sci. 2018;13:1–17. 10.1186/s13012-017-0707-4 PMC5810192 29426341

[12] Tinkle Mindy, Kimball Richard, Haozous Emily A., Shuster George, Meize-Grochowski Robin (2013). Dissemination and Implementation Research Funded by the US National Institutes of Health, 2005–2012. Nursing Research and Practice.

[13] NIH. About grants. 2017. https://grants.nih.gov/grants/about_grants.htm. Accessed 1 Sept 2016.

[14] NIH. Query form - NIH RePORTER - NIH Research Portfolio Online Reporting Tools Expenditures and Results. 2018. https://projectreporter.nih.gov/reporter.cfm. Accessed 1 Sept 2016.

[15] NIH. NIH data book - NIH Research Portfolio Online Reporting Tools (RePORT). N.D. https://report.nih.gov/nihdatabook/index.aspx. Accessed 1 Sept 2016.

[16] NIH. PAR-10-038. Dissemination and Implementation Research in Health (R01). N.D. http://grants.nih.gov/grants/guide/pa-files/PAR-10-038.html. Accessed 1 Sept 2016.

[17] Dillman D, Smyth J, Christian L (2009). Internet, mail, and mixed-mode surveys.

[18] Guest G, Namey E, Mitchell M (2013). Collecting qualitative data.

[19] Hsieh H, Shannon S (2005). Three approaches to qualitative content analysis. Qual Health Res.

[20] Flick U (2014). The Sage handbook of qualitative data analysis.

[21] Nvivo qualitative data analysis software. QSR International; 2012.

[22] Thompson C, McCaughan D, Cullum N (2004). Increasing the visibility of coding decisions in team-based qualitative research in nursing. Int J Nurs Stud.

[23] Fetters M, Curry L, Creswell J (2013). Achieving integration in mixed methods designs-principles and practices. Health Serv Res.

[24] Hanson H, Salmoni A, Volpe R (2009). Defining program sustainability: differing views of stakeholders. Can J Public Health.

[25] Kirk M, Kelley C, Yankey N, Birken S, Abadie B, Damschroder L. A systematic review of the use of the consolidated framework for implementation research. Implement Sci. 2016;11:1–13. 10.1186/s13012-016-0437-z PMC4869309 27189233

[26] Birken S, Powell B, Shea C, Haines E, Alexis Kirk M, Leeman J, Rohweder C, Damschroder L, Presseau J. Criteria for selecting implementation science theories and frameworks: results from an international survey. Implement Sci. 2017;12:1–9. 10.1186/s13012-017-0656-y PMC5663064 29084566

[27] Nilsen P. Making sense of implementation theories, models and frameworks. Implement Sci. 2015;10:1–13. 10.1186/s13012-015-0242-0 PMC4406164 25895742

[28] Chambers D, Glasgow R, Stange K. The dynamic sustainability framework: addressing the paradox of sustainment amid ongoing change. Implement Sci. 2013;8:1–11. 10.1186/1748-5908-8-117 PMC3852739 24088228

[29] CIHR. Operating grant: knowledge to action - tips from the chair and reviewers http://www.cihr-irsc.gc.ca/e/44246.html. Accessed 29 June 2018.

[30] NIHR. PAR-10-038. Invention for innovation. https://www.nihr.ac.uk/funding-and-support/funding-for-research-studies/funding-programmes/invention-for-innovation/. Accessed 29 June 2018.

