# Supplementary material for: How do researchers conceptualize and plan for the sustainability of their NIH R01 implementation projects?
Source: Implement Sci. 2019 May 9;14:50. doi: 10.1186/s13012-019-0895-1 (PMC6506963; doi:10.1186/s13012-019-0895-1)
Supplement: Supplementary file 1 — Appendix A: appendix representing the questions used for the semi-structured interview guide. (DOCX 15 kb) [file 13012_2019_895_MOESM1_ESM.docx]

**Additional file 1: Appendix A**

We designed the semi-structured interview guide below to provide interviewers with a general line of questioning. We encouraged interviewers to use their own probes to explore issues that relate to the study’s research questions. The guide was iterative and was refined throughout data collection to reflect emergent themes.

**INTERVIEW QUESTIONS**

1. How do you define sustainability when it comes to evidence based practices, programs, or strategies?
2. Tell me about your funded implementation project.
3. Have you created a plan for sustainability?
4. Have you used any frameworks to plan for or think about the sustainability of your project?

**Perceived Sustainability**

1. If complete, what is your perception of the sustainability of this project?
2. Do you have access to outcomes data on the project following formal implementation?
   1. If so, what kind of data did you collect and are there any publications that you could share with us?
3. Is there anything that the funder could have done to increase the likelihood that you would plan for sustainability?
4. Are there any other factors that could have influenced the likelihood that you would plan for sustainability?
5. Have you secured any additional funding for this project?
6. What is the future plan for this project?

**Additional Questions**

1. Do you have any other feedback, comments, or concerns regarding any of the content we have discussed today that you would like to bring up or share?

**Thank you very much for your time. Your feedback is greatly appreciated. I will stop recording now. (*turn off recorder*)**
