# Supplementary material for: How do researchers conceptualize and plan for the sustainability of their NIH R01 implementation projects?
Source: Implement Sci. 2019 May 9;14:50. doi: 10.1186/s13012-019-0895-1 (PMC6506963; doi:10.1186/s13012-019-0895-1)
Supplement: Supplementary file 2 — Appendix B: appendix representing further details on the data collection and analysis for the document review and the qualitative interviews. (DOCX 18 kb) [file 13012_2019_895_MOESM2_ESM.docx]

**Additional file 2: Appendix B**

The following are further details on the data collection and analysis for the document review and the qualitative interviews.

Document Review

*Identification*

Between September 19 and October 05, 2016, we exported data from all R01 Grants and Equivalent project profiles listed in the NIH RePORTER Database reviewed by the DIRH study section. We removed duplicate project profiles with the same grant identification number.

*Screening*

Two analysts independently screened project profiles and excluded profiles that did not have primary outcomes related to implementation. We defined implementation as the use of strategies to adopt and integrate evidence into practice within specific settings [16]. We only included projects with an implementation focus for further data abstraction and excluded those that had primary outcomes focused on dissemination. We defined dissemination as the targeted distribution of evidence to a specific public health or clinical practice audience (e.g., the comparative the effectiveness of two modes of delivering information to target audiences) [17].

*Data Abstraction*

We developed a standard template to extract data including principal investigator (PI) name, PI contact information, project start and end dates, if the project profile mentioned the term sustainability or its synonyms , sustainability definition, and sustainability plan. Two analysts independently piloted a standardized data abstraction template on 10% of NIH R01 implementation research project profiles . They held a consensus meeting to discuss any discrepancies and refine the template. After this pilot phase, they independently abstracted descriptive data from 20% of the included implementation project profiles and calculated inter-rater reliability as percent agreement. The research team held a second consensus meeting to discuss and resolve any discrepancies before continuing with data abstraction of the remaining included project profiles.

*Analysis*

We calculated descriptive statistics (e.g. proportions, means and standard deviations) for all numerical variables using Microsoft Excel.

Qualitative Interviews

*Recruitment*

In keeping with Dillman’s method [18] for respondent contact strategies we sent all PIs reminder invitations two weeks after the first invitation. We continued interviews until we reached saturation of themes, defined as the point when no new information was generated from interviews [19].

*Analysis*

We used a directed content analysis to analyze interview transcripts [20]. Two qualitative analysts recorded a memo after each interview they conducted. After they completed interviews, they used a modified coding consensus approach [21]. First, analysts collaboratively developed a draft of the coding framework based on their interview memos. Second, they used the coding framework to independently code 20% of transcripts (*n* = 2) using NVivo 11 software [22].Third, they calculated kappa coefficients to compare their coding, resolved any discrepancies through discussion, and revised the coding framework.[23] If the kappa co-efficient did not exceed 0.6, they did a second round of coding on 20% (*n* = 2) of transcripts. Once the kappa co-efficient exceeded 0.60, the remaining transcripts were coded by a single analyst.
