# Supplementary material for: How do researchers conceptualize and plan for the sustainability of their NIH R01 implementation projects?
Source: Implement Sci. 2019 May 9;14:50. doi: 10.1186/s13012-019-0895-1 (PMC6506963; doi:10.1186/s13012-019-0895-1)
Supplement: Supplementary file 3 — Appendix C: appendix representing the frequency of sustainability synonyms used across R01 DIRH implementation project profiles. (DOCX 14 kb) [file 13012_2019_895_MOESM3_ESM.docx]

**Additional file 3: Appendix C**

Frequency of sustainability synonyms used across R01 DIRH implementation project profiles

| **Synonym/Variant** | **Frequency (n)** | **Percentage (%)** |
| --- | --- | --- |
| Sustainability | 22 | 22.9 |
| Sustainable | 9 | 9.4 |
| Sustainment | 8 | 8.3 |
| Sustain | 8 | 8.3 |
| Sustained | 19 | 19.8 |
| Sustaining | 5 | 5.2 |
| Maintenance | 14 | 14.6 |
| Maintaining | 3 | 3.1 |
| Maintain | 2 | 2.1 |
| Continue | 5 | 5.2 |
| Long term | 1 | 1.0 |
| **Total** | 96 | 100 |
